# The *Trypanosoma cruzi* Vitamin C Dependent Peroxidase Confers Protection against Oxidative Stress but Is Not a Determinant of Virulence

**DOI:** 10.1371/journal.pntd.0003707

**Published:** 2015-04-13

**Authors:** Martin C. Taylor, Michael D. Lewis, Amanda Fortes Francisco, Shane R. Wilkinson, John M. Kelly

**Affiliations:** 1 Faculty of Infectious and Tropical Diseases, London School of Hygiene and Tropical Medicine, London, United Kingdom; 2 School of Biological and Chemical Sciences, Queen Mary University of London, London, United Kingdom; Universidad Autónoma de Yucatán, MEXICO

## Abstract

**Background:**

The neglected parasitic infection Chagas disease is rapidly becoming a globalised public health issue due to migration. There are only two anti-parasitic drugs available to treat this disease, benznidazole and nifurtimox. Thus it is important to identify and validate new drug targets in *Trypanosoma cruzi*, the causative agent. *T*. *cruzi* expresses an ER-localised ascorbate-dependent peroxidase (TcAPx). This parasite-specific enzyme has attracted interest from the perspective of targeted chemotherapy.

**Methodology/Principal Findings:**

To assess the importance of TcAPx in protecting *T*. *cruzi* from oxidative stress and to determine if it is essential for virulence, we generated null mutants by targeted gene disruption. Loss of activity was associated with increased sensitivity to exogenous hydrogen peroxide, but had no effect on susceptibility to the front-line Chagas disease drug benznidazole. This suggests that increased oxidative stress in the ER does not play a significant role in its mechanism of action. Homozygous knockouts could proceed through the entire life-cycle *in vitro*, although they exhibited a significant decrease in their ability to infect mammalian cells. To investigate virulence, we exploited a highly sensitive bioluminescence imaging system which allows parasites to be monitored in real-time in the chronic stage of murine infections. This showed that depletion of enzyme activity had no effect on *T*. *cruzi* replication, dissemination or tissue tropism *in vivo*.

**Conclusions/Significance:**

TcAPx is not essential for parasite viability within the mammalian host, does not have a significant role in establishment or maintenance of chronic infections, and should therefore not be considered a priority for drug design.

## Author Summary

The single-cell parasite *Trypanosoma cruzi* causes Chagas disease, one of the major neglected tropical infections. Throughout Latin America, this disease is an important cause of premature death due to heart failure, and it is currently an emerging public health problem in the US, Europe and across the world. There are only two drugs available to treat the infection, benznidazole and nifurtimox. Both have significant side effects and resistance/treatment failures are increasing. The *T*. *cruzi* vitamin-C dependent peroxidase (TcAPx) is an anti-oxidant enzyme which is absent from the mammalian host, and has been proposed as a potential drug target. Using genetic manipulation, we have deleted the genes for this enzyme from *T*. *cruzi*. The resulting null mutants were hypersensitive to hydrogen peroxide but displayed normal sensitivity to benznidazole. Using a bioluminescent model of infection, we could demonstrate that the null mutants were able to establish a similar infection to that of wild type parasites. Our results show that TcAPx should not be pursued as a target for drug design.

## Introduction

The protozoan parasite *Trypanosoma cruzi* is the causative agent of Chagas disease. In Latin America, 8–10 million people are infected, with many more at risk. In addition, as a result of migration, the disease is becoming a public health issue in non-endemic regions, such as Europe and the US [1–3]. Infection with *T*. *cruzi* is usually life-long, and up to 30% of individuals develop chronic Chagas disease, with symptoms that include cardiomyopathy and/or digestive megasyndromes. Treatment of *T*. *cruzi* infection is dependent on two drugs first introduced in the 1970s, benznidazole and nifurtimox. Both of these nitroheterocyclic compounds can have toxic side effects and do not consistently result in sterile cure, particularly in adults. Whilst benznidazole is curative in the acute stage of the disease [4], its efficacy in the chronic phase remains controversial, despite much research effort [5, 6]. A further problem which impacts on the widespread use of benznidazole and nifurtimox is the potential for cross-resistance. Both compounds are pro-drugs and are activated within the parasite by the same mitochondrial nitroreductase (TcNTR). Activation of benznidazole results in depletion of the cellular thiol pool, likely leading to a reduced ability to deal with oxidative stress [7]. Loss of, or mutations within *TcNTR*, can result in resistance to both of the front-line drugs [8–10]. Consequently, there is an urgent need for new chemotherapeutic agents.

Many aspects of trypanosome biochemistry are distinct from their mammalian hosts, and as such, have been proposed as targets for drug design. One example is the *T*. *cruzi* vitamin C dependent hemoperoxidase TcAPx, an enzyme belonging to Class 1 of the peroxidase–catalase superfamily [11]. This group of enzymes, which are absent from mammals, has been reclassified to separate true ascorbate peroxidases (APx) and cytochrome c peroxidases (CcP) from the hybrid type A and B APx-CcP groups, which show characteristics of both [12]. TcAPx falls into the hybrid type A group, which includes APx from the closely related *Euglena gracilis*, and from algae and oomycetes. The function of APx enzymes is to reduce H_2_O_2_ to H_2_O using ascorbate as an electron donor, thereby minimising the production of highly reactive hydroxyl radicals. APxs are particularly important in photosynthetic plants which contain isoforms targeted to each cellular compartment where reactive oxygen species (ROS) are formed. For example, plastid targeted isoforms protect the plant from H_2_O_2_ generated during photosynthesis [13].

In trypanosomatids there is only one APx isoform, which in *T*. *cruzi* is targeted to the endoplasmic reticulum (ER) [11]. The major source of H_2_O_2_ in the ER is oxidative protein folding, a process mediated by enzymes such as the flavoprotein ER oxidoreductin (Ero1). Ero1 uses molecular oxygen to oxidise protein disulphide isomerase, the enzyme required for disulphide bridge formation in the ER. For each disulphide bond generated, a molecule of H_2_O_2_ is formed, and this process can therefore generate high levels of oxidative stress. In the ER, TcAPx has the capacity to prevent this by reducing the resulting H_2_O_2_ before it builds to toxic levels. APx is also found in the related parasite *Leishmania*, where it is a mitochondrial enzyme [14], and in several other kinetoplastids, but it is absent from the African trypanosomes, *Trypanosoma brucei*, *Trypanosoma congolense* and *Trypanosoma vivax* [15].

The importance of TcAPx to the viability and infection potential of *T*. *cruzi* is unknown. Proteomic studies have suggested that there is increased expression in the infectious metacyclic trypomastigote forms [16]. However, it has also been shown that TcAPx expression levels are not related to virulence or metacyclogenesis in a panel of ten parasite strains, whereas expression of other antioxidant enzymes (the mitochondrial and cytosolic peroxiredoxins) does correlate with infectivity [17]. Here, we describe a series of experiments designed to determine whether the parasite-specific TcAPx enzyme has a crucial role in the infection process.

## Methods

### Parasite culture

*T*. *cruzi* epimastigotes (strain Sylvio X10/6) were maintained in RPMI-1640 supplemented as previously described [18] at 27°C. L6 rat myoblast and Vero cells were cultured in the same medium but without hemin and trypticase, at 37°C in 5% CO_2_. Metacyclic parasites were obtained from stationary phase epimastigote cultures as previously reported [9]. Mammalian cell monolayers were infected by addition of metacyclic trypomastigotes at a ratio of 5:1 (parasites:host cells). Parasite transfection was carried out using an Amaxa Nucleofector II device with human T-cell buffer (Lonza). 5 x10^7^ epimastigotes were transformed with 5–10 μg of construct DNA. Drug selection was carried out at 10 μg ml^-1^ blasticidin, 5 μg ml^-1^ puromycin, 100 μg ml^-1^ G418 and 150 μg ml^-1^ hygromycin, as appropriate (InVivoGen). Parasite cloning was carried out by diluting the parasite suspension to a concentration of 2 cells ml^-1^ and plating in 96-well microtitre plates at 100 μl per well. Plates were maintained at 27°C in 5% (v/v) CO_2_ with humidity.

### Production of TcAPx antibodies

Recombinant his-tagged TcAPx was purified as described [11]. The protein was electrophoresed on 10% (w/v) SDS-PAGE gels and the TcAPx band excised. This was frozen in liquid nitrogen, ground to a fine powder, mixed with Freund’s complete adjuvant, and injected into mice. A pre-immunisation serum sample was obtained prior to injection. Antibodies were tested by western blotting against recombinant TcAPx and trypanosome lysates. Membranes were probed with mouse anti-TcAPx (1:1000), followed by goat anti-mouse HRP conjugate (BioRad), and developed using ECL+ kit (GE Healthcare). Protein concentrations were assayed using the BCA method (Pierce).

### Construction of targeting vectors

For gene disruption constructs, the *TcAPx* ORF (TcCLB.506193.60) was amplified from *T*. *cruzi* genomic DNA and cloned into pGEM-T easy (Promega) using primers 5’-CAGGCAAGGTACCGTTTTCTTCAT and 5’-TTTTGACTCTGCTGGGAGAG. The *TcAPx* insert was then isolated with Sac I and Sph I and sub-cloned into Sac I/Sph I digested pUC19 to create pUC-TcAPx-2. The plasmid pUC-TcAPx-2 was cut with Nae I and Apa I resulting in the deletion of the central 284 bp from *TcAPx*. Drug resistance cassettes for puromycin and blasticidin were ligated into this gap to produce the disruption constructs pTcAPx-Δ284-PAC and pTcAPx-Δ284-BLA. The resistance cassettes used RNA processing signals from *T*. *brucei* tubulin.

The gene deletion construct pTcAPx-KO-BLA was constructed as follows. The 5’ flank encompassing the 3’ end of the *CLPTM1* gene (TcCLB.506193.20) and the intergenic sequence up to centre of the *TcAPx* ORF, was amplified from genomic DNA using primers 5’-AATCCATCGTCTCTTGAAT and 5’-CTTGAGCGATTCCAGCGCA. The template DNA was isolated from the *TcAPx* ^+/-^ cell line to ensure that the amplicon was specific to the second intact *TcAPx* allele to allow efficient targeting. The 3’ end encompassing the STOP codon of *TcAPx*, the downstream intergenic sequence and the 5’ end of the *G6PDH* gene (TcCLB.506193.70) was amplified using primers 5’-TGACGCGTCCAGGTGCAG and 5’-TTGCACCGAGTACCACGAT. The 3’ flank was cloned into pGEM-T to produce p*TcAPx*-3’flank. The BLA drug resistance cassette was cloned as a Not I /blunted Apa I fragment into Not I/Sma^TM^ I cut p*TcAPx*-3’flank to produce pBLA-3’KO. The 5’ flank was cloned into pGEM-T then isolated as a Not I/Bam HI fragment, which was cloned into Not I/Bam HI cut pBLA-3’KO to produce p*TcAPx*-KO-BLA. For transfection the fragment was isolated following Not I/Apa I digestion. Construction of the episomal expression vector pTEX-APx is described elsewhere [11].

The luciferase reporter construct pTRIX2-RE9h [19] was modified for bioluminescent tagging of null mutants by removal of the *Neo* ^R^ gene and its replacement with a *Hyg* ^R^ gene to generate pTRIX2-RE9h-Hyg.

### Susceptibility testing

Parasites in the logarithmic phase of growth were diluted back to 5 x 10^5^ ml^-1^ in 96-well plates. The appropriate concentration of drug was added and the plates were incubated at 27°C. Each drug concentration was tested against each cell line in quadruplicate. Resazurin (Sigma) was added after 5 days and the plates incubated for a further 4 days. 0.1% SDS was added to each well to lyse the parasites and the plates then read in a Spectramax M3 Microplate reader. Results were analysed using GraphPad Prism.

### Cell invasion assays

L6 rat myoblasts or Vero cells were plated in chamber slides. They were allowed to settle for 16 hours and then infected with metacyclic trypomastigotes at a ratio of 5 trypanosomes per cell. The infection was incubated for 48 hours at 37°C, then extracellular trypanosomes removed by extensive washing in serum-free medium. After washing, slides were fixed in 100% methanol at room temperature for 15 minutes. The chamber walls were removed and the cells stained with Giemsa. The proportion of cells carrying intracellular parasites was calculated as a measure of infectivity. Seven replicates were performed per infection.

### Luciferase activity assay

Parasites from an exponentially growing culture were counted, pelleted and washed in PBS. They were lysed in Cell Culture Lysis Reagent (CCLR, Promega). Luciferase activity was measured using the luciferase assay system (Promega) according to manufacturer’s instructions. Cell extracts were diluted as necessary in CCLR supplemented with 100 μg ml^-1^ bovine serum albumin. Each assay was performed on two individual extracts per cell line and in duplicate per extract. Luminescence was monitored on a SpectraMax M3 Microplate Reader (Molecular Devices GmbH).

### Mouse infection studies

All animal work was carried out under UK Home Office project licence (PPL 70/6997) and was approved by the London School of Hygiene and Tropical Medicine Animal Welfare and Ethical Review Body. All protocols and procedures were conducted in accordance with the UK Animals (Scientific Procedures) Act 1986 (ASPA). Animals were maintained under specific pathogen-free conditions in individually ventilated cages. They experienced a 12 hour light/dark cycle and had access to food and water *ad libitum*.

Female BALB/c mice aged 8–12 weeks (Charles River UK) were infected by intra-peritoneal injection with 2 x 10^5^ culture-derived trypomastigotes. The course of infection was monitored by bioluminescent imaging as detailed elsewhere [19]. Briefly, 10 minutes prior to imaging, mice were injected i.p. with 150 mg kg^-1^ d-luciferin in Dulbecco’s modified PBS. They were anaesthetised with 2.5% (v/v) isoflurane in oxygen, then placed in the IVIS Illumina II system (Caliper Life Sciences). Images were acquired using Living Image 4.3 software with an exposure time of up to 5 minutes. After imaging, mice were weighed and revived, then placed back into their cages. For *ex-vivo* imaging, mice were injected with d-luciferin as above, then terminally anaesthetised with Euthatal (Merial) and sacrificed by exsanguination. Mice were perfused with 10 ml 0.3 mg ml^−1^ d-luciferin in Dulbecco’s modified PBS via the heart. Organs were removed, placed on a Petri dish and soaked in 0.3 mg ml^−1^ d-luciferin, then imaged as previously described [19]. All imaging data were analysed with Living Image^TM^ 4.3 software (Caliper Life Sciences), using uninfected animals to set the base line for background luminescence.

### Statistical analyses

Statistical analysis of differences between groups or values was carried out using Student’s t-test, the F-test or one-way ANOVA depending on the experiment. Figure legends indicate the test used in each experiment. All analysis was processed using GraphPad Prism software.

## Results

### Generation of TcAPx null mutants

**Fig 1:**
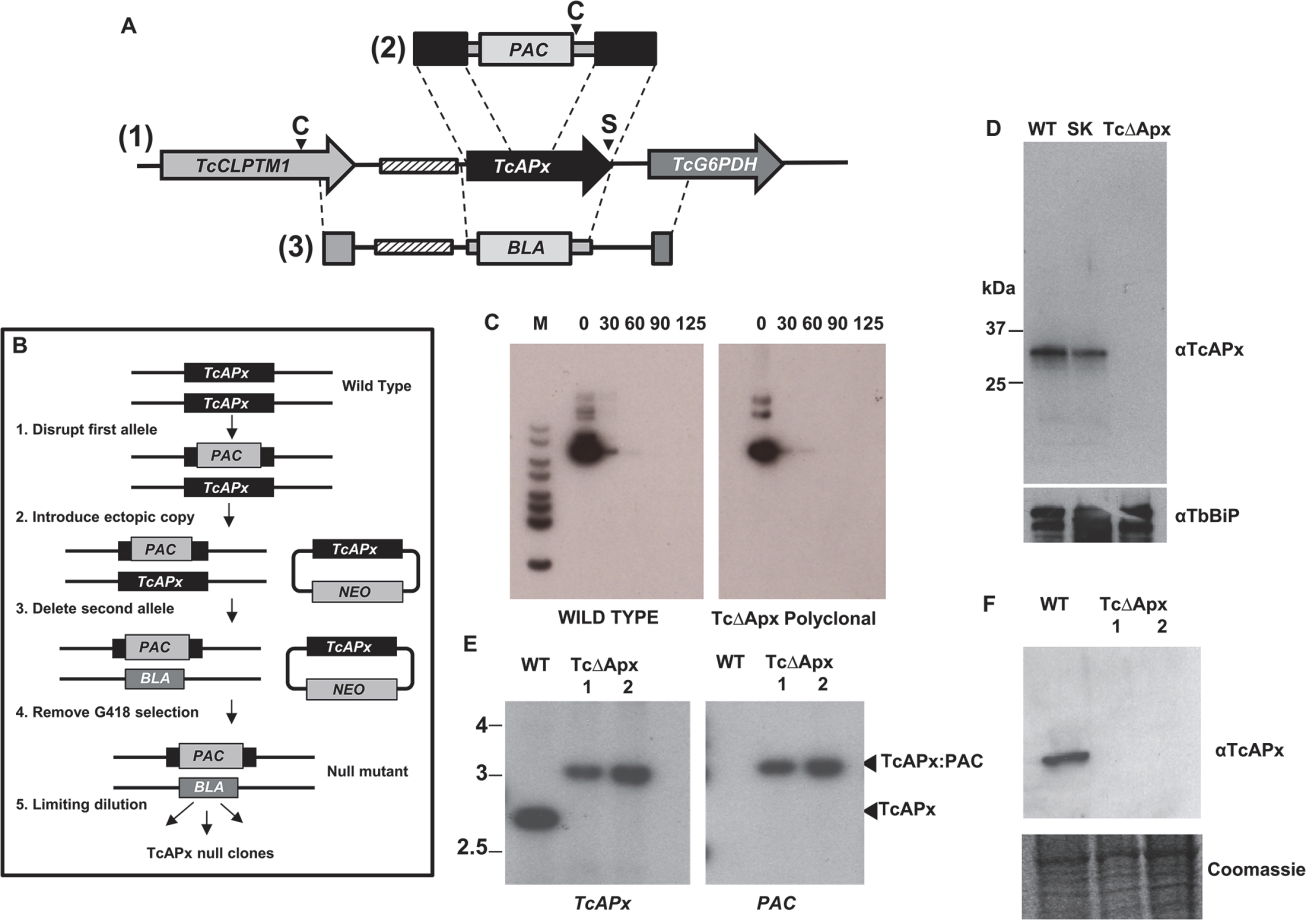
Generation of *TcAPx* null mutants. **A** Map of the *T*. *cruzi* Sylvio X10/6 *TcAPx* locus (1) indicating the derivation of constructs used for targeted integration. The location of the flanking *TcCLPTM1* (Cleft-lip and palate transmembrane 1–like protein) and *TcG6PDH* (glucose-6-phosphate dehydrogenase) genes are indicated and the hatched box represents a degenerate VIPER/SIRE element. The ‘first round’ gene disruption construct is shown in (2), with the ‘second round’ gene deletion vector represented by (3). Restriction sites shown are C: Cla I and S: Sma I. **B** Strategy used to generate TcAPx null mutants. Briefly, the first allele was disrupted by insertional integration of the *PAC* gene into the ORF (1). An episomal copy of TcAPx was introduced into the *TcAPX* ^+/-^ heterozygote line (2). The second endogenous allele was then deleted by homologous recombination using the flanking DNA external to the ORF to insert the *BLA* gene (3). The parasites were then removed from G418 selective pressure and passaged for up to 125 generations (4). Clones were then isolated and characterised (5). **C** The pTEX-APx episome is unstable in both wild type and *TcAPx* null backgrounds. The autoradiographs show Southern blots containing genomic DNA from wild type and null mutant cells isolated before (lanes 0) and after removal of G418 from the growth medium. Generations without G418 selection are indicated above the blot. The blot was probed with the *Neo* ^R^ ORF. **D** Western blot showing expression level of TcAPx in parasite populations 125 generations after removal of G418 selection. WT: wild type Sylvio X10/6 lysate; SK: lysate from cells with a single copy of *TcAPX* disrupted; TcΔAPx: lysate from cells with both genes ablated. The panel below shows the blot probed with anti-TbBiP (a kind gift from Jay Bangs, University of Wisconsin-Madison) to control for loading. **E** Southern blot of genomic DNA digested with Cla I and Sma I, showing that *TcAPx* is absent from cloned null mutant cells. The left hand panel was probed with the *TcAPx* ORF and shows the endogenous gene (lane WT, 2.8 kb Cla I-Sma I fragment) and the gene disrupted by *PAC* insertion (lanes TcΔAPx 1 and 2, 3.1 kb Cla I fragment). **F** Western blot indicating that the null mutant clones do not express TcAPx. The wild type population show a single band of ~30kDa (lane WT: wild type) which is absent from the null mutant clones (lanes TcΔAPx 1 and 2). Equivalent loading is indicated by the Coomassie stained gel below.

The *TcAPx* loci are found on chromosome 36 of the *T*. *cruzi* genome reference strain CL-Brener (TcVI group). In this hybrid lineage, there are considerable organisational differences in the structure of the loci between the Esmeraldo-like (EL, TcII derived) and non-Esmeraldo-like (NEL, TcIII derived) haplotypes (S1 Fig), indicative of extensive rearrangement. For gene deletion studies, we therefore selected the Sylvio X10/6 strain (TcI group), where the organisation of these loci is conserved between chromosome homologues. Consecutive rounds of targeted gene disruption were undertaken to test the feasibility of generating *TcAPx* null mutants (Fig 1A, Methods). We could readily disrupt a single allele, but it was not possible to ablate both copies of the gene, despite multiple attempts. Either a third allele was detected after selection, the second construct recombined with the modified allele, or the drug resistance cassette formed episomes made up of head-to-tail tandemly repeated copies (S2 Fig).

Failure to achieve sequential disruption of both gene copies is often considered as evidence that the encoded protein has an essential function. To explore this further, we attempted to delete the second copy of the gene in epimastigotes which had been modified to express TcAPx from an episome (Fig 1B and 1C, pTEX-APx described in [11]). Transformants were obtained following transfection with the integrative vector and the absence of the second endogenous *TcAPx* allele confirmed by Southern blotting. By implication, failure to generate null mutants in the absence of an ectopic copy did not arise from off-target effects of the gene inactivation process.

To determine if *TcAPx* is an essential gene, we cultured two individually derived populations of the complemented, homozygous deletion mutants in the absence of G418, the selective drug required for maintenance of the episome. In *T*. *cruzi*, episomes undergo random segregation. Therefore, in cells where both chromosomal copies of *TcAPx* had been disrupted, loss or retention of pTEX-APx, in the absence of selective drug pressure should reveal if the gene is essential. A similar technique has been developed for testing essentiality in *Leishmania* [20]. Analysis of parasite DNA prepared after the removal of drug selection revealed that the episome was lost from the population with similar kinetics to wild type cells transformed with pTEX-APx episome (Fig 1C). Using the *Neo* ^*R*^ gene as a probe, it was apparent that the copy number had fallen by >90% within 60 generations (approximately 60 days) and to undetectable levels after 90. Western blotting confirmed that the TcAPx protein was no longer present at detectable levels (Fig 1D). For phenotypic analysis, clonal lines were derived from each of these populations (Methods). These were negative for both the endogenous and ectopic copies of *TcAPx* (Fig 1E, the 3.1 kb Cla I hybridising band present in the null mutant lanes corresponds to the disrupted copy of the gene as shown by hybridisation with the *PAC* probe; see map Fig 1A) and did not express the protein (Fig 1F).

**Fig 2:**
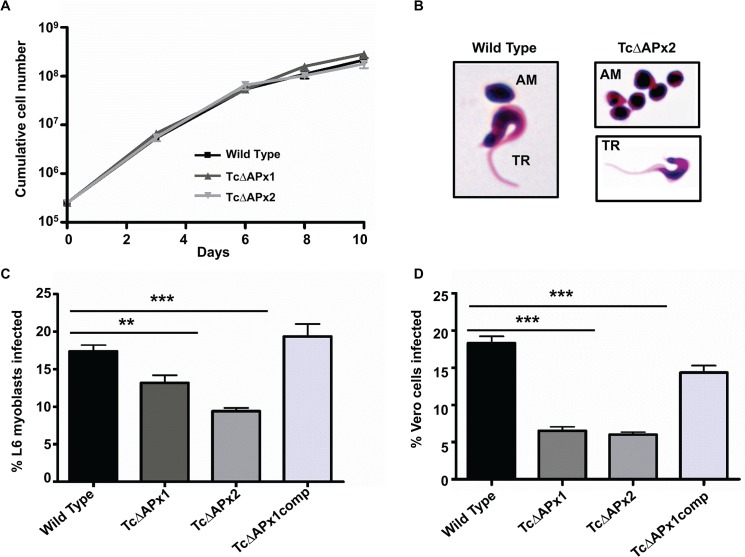
Phenotypic assessment of null mutants *in vitro*. **A** Growth rate of *T*. *cruzi* epimastigotes for wild type and null mutant (TcΔAPx1 and 2) clones. Triplicate cultures were followed for 10 days. There was no significant difference in growth rate. **B** Parasites lacking TcAPx can differentiate to amastigotes (AM) and trypomastigotes (TR). Examples shown are Giemsa stained wild type and null mutant (TcΔAPx2) cells. **C** *In vitro* infectivity for L6 rat myoblast cells. Metacyclic trypomastigotes were used to infect L6 cells at a ratio of 5 trypanosomes per cell and left for 48 hours (Methods). Cells were Giemsa stained and the number of infected cells counted. Infections were carried out with seven replicates per parasite line. TcΔAPx1comp refers to TcΔAPx1 cells retransformed with pTEX-APx to complement the null phenotype. Data presented as mean + SD. Significance of difference between each pair was assessed by Student’s t-test, (**) corresponds to *P* = 0.007, (***) *P* = 0.0006. *P* values for wild type:TcΔAPx1 indicated by short horizontal line, wild type:TcΔAPx2 indicated by long horizontal line. The difference between the wild type and complemented lines was not significant. **D** *In vitro* infectivity for Vero epithelial cells. Metacyclic trypomastigotes were used to infect Vero cells at a ratio of 5 trypanosomes per cell and left for 48 hours (Methods). Cells were Giemsa stained and the number of infected cells counted. Infections were carried out with seven replicates per parasite line. Data presented as mean + SD. Significance of difference was assessed by Student’s t-test, (***) corresponds to *P* = 0.0006. *P* values for wild type:TcΔAPx1 indicated by short horizontal line, wild type:TcΔAPx2 indicated by long horizontal line.

The null mutants showed no obvious growth phenotype when cultured as epimastigotes (Fig 2A) and could differentiate into metacyclic trypomastigotes in stationary phase cultures. These metacyclic trypomastigotes were able to infect L6 cells, a rat myoblast line, and Vero cells, albeit at a somewhat reduced level (Fig 2C and 2D). Once inside the host cell, they were able to differentiate into amastigotes (Fig 2B). The amastigotes differentiated to bloodstream trypomastigotes and lysed the cells as normal. The released trypomastigotes were fully capable of differentiating back into epimastigotes or re-infecting naïve cells. Thus, the null mutants could complete the entire life-cycle *in vitro*.

### Susceptibility of TcAPx null mutants to H_2_O_2_ and benznidazole

**Fig 3:**
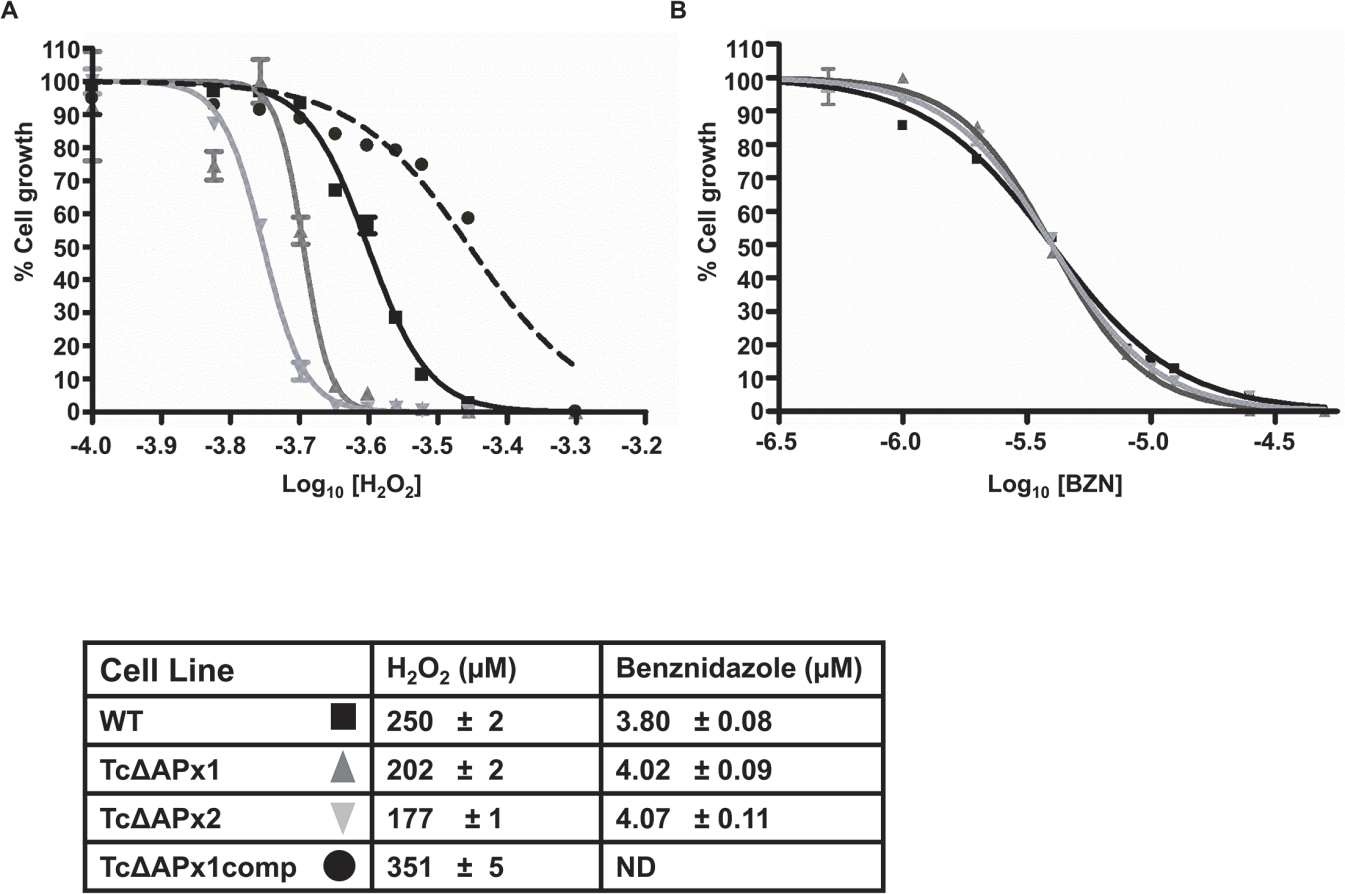
*In vitro* sensitivity of null mutants to oxidative stress and benznidazole. Epimastigotes seeded at 5 x 10^5^ ml^-1^ were exposed to various concentrations of **A** hydrogen peroxide and **B** benznidazole. The number of viable cells after 9 days was measured using resazurin fluorescence. TcΔAPx1comp refers to TcΔAPx1 cells retransformed with pTEX-APx to complement the null phenotype. Data were analysed by sigmoidal curve fitting using GraphPad Prism. The table below shows the EC_50_ values (μM) for each compound against the various cell lines +/- standard deviation. Significance of differences in the EC_50_ for H_2_O_2_ was measured using the F-test. (ND—not done).

Parasites which over-express TcAPx are more resistant to exogenous H_2_O_2_ than wild type (Wilkinson et al., 2002a), implying that cells lacking this enzyme might be hypersensitive. This proved to be the case, with the null mutants showing a significant fall in their EC_50_ values (Fig 3A) (*P*<0.0001). Reintroduction of an ectopic copy of the gene decreased H_2_O_2_ susceptibility to a level above that exhibited by wild type parasites, indicating that the sensitivity phenotype was due to the loss of *TcAPx*. It can be inferred that the higher EC_50_ value displayed by the complemented cell line results from the enhanced *TcAPx* expression level in epimastigotes containing a multicopy episome, as demonstrated previously in a wild type background [11].

The front line drug used to treat Chagas disease was also tested against the null mutants (Fig 3B). Benznidazole is activated in the trypanosome mitochondrion by the action of a type I nitroreductase to produce toxic metabolites [21]. The compartment(s) in which these metabolites mediate their trypanocidal effect(s) are unknown, as is their final target(s)/mode of action. Metabolomic studies have suggested that benznidazole biotransformation has a major effect on thiol biochemistry within the parasite, leading to significant depletion of the major low molecular weight thiol, trypanothione [7]. Trypanothione is a key mediator of electron transfer to components of the antioxidant defence system, including TcAPx [22]. Previous work has also suggested that overexpression of Fe-superoxide dismutase increased susceptibility to benznidazole in *T*. *cruzi* [23]. Thus, one possibility is that benznidazole-mediated toxicity could result from depletion of antioxidant defences after activation by TcNTR. However, when we examined drug-sensitivity, there was no statistically significant difference between the EC_50_ values for wild type and *TcAPx* null mutants with benznidazole (Fig 3B). Thus ablation of TcAPx activity does not increase susceptibility to benznidazole or its metabolites. This is consistent with a previous observation which reported that increased TcAPx expression does not confer benznidazole resistance [11]. Therefore, if redox stress has any role in the activity of this drug, it is unlikely that generation of H_2_O_2_ within the ER is a significant component.

### Ability of the TcAPx null mutant parasites to infect mice

The null mutants retained an ability to progress through the life cycle *in vitro* but did display a slightly decreased ability to infect cultured mammalian cells and an increased sensitivity to H_2_O_2_. We therefore investigated whether these deleterious phenotypes had any effect on their ability to establish a chronic infection in a murine model. The Sylvio X10/6 strain used in this study is not highly virulent. In most murine models, parasites are rarely detected in the bloodstream by microscopy, even during the acute stage of infection. This has been noted with other clones in the Sylvio X10 series [24]. We therefore exploited a highly sensitive *T*. *cruzi* bioluminescence imaging model developed in our laboratory, which allows chronic infections to be monitored in real time [19]. In this model, there is a linear relationship between parasite burden and bioluminescence, and a robust correlation with qPCR. Both wild type and TcΔAPx null mutant parasites were transformed with the pTRIX2-RE9h-Hyg vector (Methods), which facilitates the targeting of a red-shifted luciferase gene [25] into the RRNA array, such that expression is under the control of a strong RNA polymerase I dependent promoter.

The growth rate of the bioluminescent transfectants was assayed *in vitro* to determine whether expression of the luciferase gene had any effect. The doubling time was not significantly different between the wild type pTRIX2-Re9h-Hyg transformants and the *TcAPX* null mutants expressing luciferase (S3 Fig). The bioluminescent lines were also assayed for luciferase activity to ensure that the clones used in infection studies expressed similar levels of bioluminescence. The luciferase activity for each of the cell lines is shown in S4 Fig.

**Fig 4:**
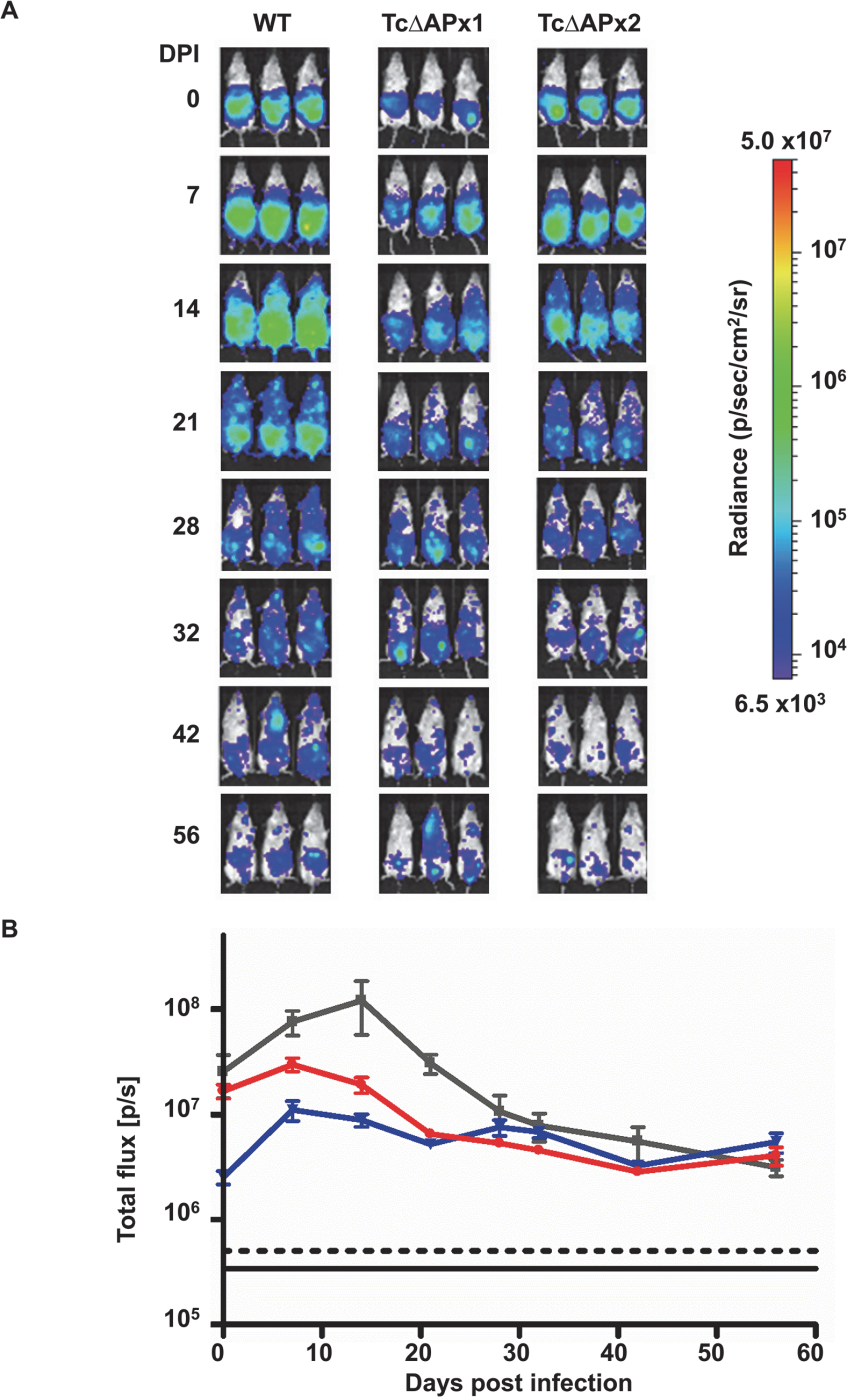
Course of infection in a murine model monitored by bioluminescence imaging. **A** Female BALB/c mice were infected with 2 x 10^5^ culture-derived bloodstream trypomastigotes modified to express a red-shifted luciferase gene (Methods, [19]). Mice were imaged at the time points shown using an IVIS Illumina II system (Caliper Life Sciences). Images were analysed using the same signal intensity scale for radiance (right) where purple indicates low signal intensity and red indicates a high signal. The maximum (5x10^7^) and minimum (6.5 x10^3^) signals are indicated at the top and bottom of the scale bar respectively. Three representative mice are shown from each group of animals (*n* = 6 per group). DPI: days post infection, time 0 represents image taken one hour after infection. **B** Graph showing the mean total body flux measured in each group of animals throughout the experiment. The grey line indicates the wild type infection, blue is TcΔAPx1 and red is TcΔAPx2. Data are plotted as mean values, error bars indicate standard deviation. The black lines indicate the mean (solid line), and mean +2SD (dotted line) of background luminescence of control uninfected mice. All data were acquired and analysed using Living Image software (Caliper Life Sciences).

BALB/c mice were inoculated with bioluminescent trypanosomes (Methods) and the course of infection followed over 56–60 days. Six mice were infected with each parasite line. Both null mutant clones showed a similar pattern of infection to the bioluminescent wild type cells (Fig 4A). There was an initial dispersal of parasites from the intra-peritoneal injection site, with dissemination throughout the mice during the 14 days leading to the peak of the acute stage of the wild type infection. With the Sylvio X10/6 strain the wild type does not produce a symptomatic, patent acute phase and trypomastigotes are not observed in blood smears. This has also been demonstrated with the Sylvio X10/4 strain [24]. The null mutants showed a similar pattern of dispersal throughout the mouse, although total body flux peaked between day 7 and 14 rather than at day 14 (Fig 4B). By day 28 all three cell lines were behaving comparably, and a more focal pattern of infection, characteristic of the chronic stage, was observed (Fig 4A). The total body flux suggested very similar levels of parasite burden after day 28 (Fig 4B), regardless of the presence or absence of TcAPx This infection profile matches that seen with the CL-Brener strain [19].

**Fig 5:**
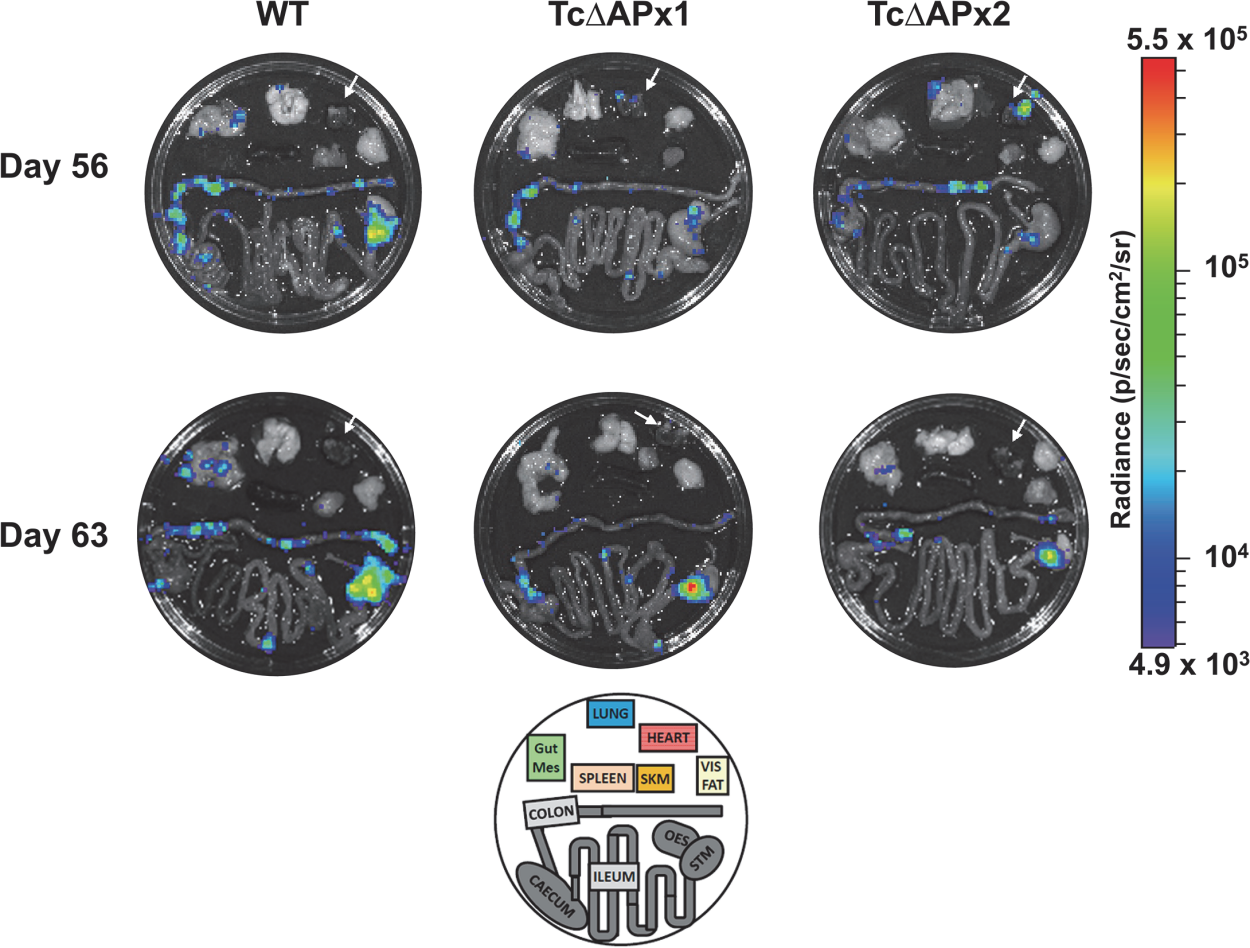
The gastrointestinal tract is the major site of *T*. *cruzi* persistence in mice infected with either wild type or null mutant parasites. *Ex vivo* imaging of organs from two representative mice of each group (groups as in Fig 4), one sacrificed at day 56 (**A**) and one at day 63 (**B**). Each of the images is on the same luminescence scale (right) for radiance where purple indicates low signal intensity and red indicates a high signal. The maximum (5.5x10^5^) and minimum (4.9 x10^3^) signals are indicated at the top and bottom of the scale bar respectively. The heart is indicated in each image by a white arrow. Foci of infection are visible in the stomach and colon in all cases. Occasional foci were detected in the heart, gut mesenteries and lungs. The schematic at the bottom of the TcΔAPx1 column indicates the layout of organs in each dish, abbreviations: Gut Mes: Gut mesenteries, SKM: skeletal muscle (right hind leg), vis fat: visceral fat, OES: oesophagus, STM: stomach.

*Ex vivo* imaging of selected tissues and organs from necropsies of infected mice immediately *post-mortem* (Methods) showed that the gastro-intestinal tract (stomach and/or colon) was the major site of parasite persistence following establishment of chronic stage infection. Sporadic bioluminescent foci were observed associated with other sites in some animals, including the gut mesenteries, heart and lungs, but there was no pattern with respect to experimental groups (Fig 5). Thus, there were no significant differences in tissue-specific distribution observed between the wild type parasites and the null mutants. This profile of persistence in the GI tract and sporadic infection of other sites is also observed with the CL-Brener strain at a similar stage of infection [19]. Taken together, these results therefore indicate that TcAPx is not essential for the establishment of an acute infection, dispersal of parasites throughout the host, or for their persistence in their gastro-intestinal niche during the chronic stage.

## Discussion

In this study, we have shown that the *T*. *cruzi* ascorbate peroxidase protects the parasite from H_2_O_2_ exposure, but it is dispensable during each life-cycle stage *in vitro* and is not required to give rise to chronic infections in a mouse model. Initially, we had found that sequential targeted disruption of both *TcAPx* alleles could not be achieved, except in the presence of an episomal copy. However, further experimentation demonstrated that this ectopic copy itself was not maintained in the absence of selective (G418) pressure, and that null mutants were then obtained. It is reasonable to assume that total loss of TcAPx activity must have detrimental consequences to the parasite under the conditions pertaining during the selection process, and that this is sufficient to prevent the outgrowth of homozygote knockouts. The subsequent ability of null mutants to survive the gradual loss of the ectopic copies could reflect differences in the culture environment in the two situations, and/or a metabolic adaptation over time which accommodates the loss of ascorbate peroxidase activity. This outcome highlights the fact that the inability to generate *T*. *cruzi* null mutants by conventional methods should not, of itself, be taken as evidence that a gene is essential.

Our data imply that TcAPx activity is not required for any of the fundamental processes governing parasite replication, development and virulence. The enzyme is localised to the endoplasmic reticulum and, unlike other trypanosomatid peroxidases, it has a substrate specificity that is limited to H_2_O_2_ [11, 26]. In the ER, H_2_O_2_ is produced as part of the reaction cycle of Ero1, a luminal membrane associated flavoprotein that mediates disulphide bond formation in client proteins, with one molecule of H_2_O_2_ produced each time Ero1 reduces protein disulphide isomerase [27, 28]. In mammalian cells which lack APx, the ER resident glutathione peroxidases 7 (GPx7) and 8 (GPx8) are utilised to remove H_2_O_2_ generated during this process [29, 30]. Trypanosomatids do not have selenium-dependent glutathione peroxidases such as GPxs 7 and 8. It is likely therefore, that a major role of TcAPx in *T*. *cruzi* is to eliminate peroxide molecules produced by the Ero1 reaction. In *T*. *brucei* and *Leishmania*, which lack an ER-localised APx, this function may be performed by other ER associated peroxidases [31], or the H_2_O_2_ may itself be utilised as an oxidant in protein folding as has been shown in mammalian cells [30, 32].

The membrane-permeable properties of H_2_O_2_ allow it to penetrate all compartments of the cell, including the ER. This is evidenced by our observation that overexpression of TcAPx confers protection against exogenous H_2_O_2_ exposure, whereas depletion results in enhanced sensitivity. Thus, our results suggest that in the null mutants, there are no effective alternative mechanisms for clearing high levels of H_2_O_2_ from the ER, and that this leads to cell death at lower exogenous concentrations than in wild type parasites. As Ero1 is localised in the ER, it could be that membrane phospholipids are the primary target of the H_2_O_2_ generated by this protein. *T*. *cruzi* also expresses an ER resident non-selenium glutathione dependent peroxidase (TcGPX II) which catalyses the reduction of lipid hydroperoxides [26]. This activity may compensate to an extent for depletion of TcAPx by protecting the ER membrane from oxidative damage. ER resident ascorbate itself may also have an additional antioxidant effect, even in the absence of TcAPx.

The susceptibility of null mutants to benznidazole was the same as wild type parasites. This is consistent with previous data which showed that overexpression of TcAPx has no effect on susceptibility to this nitroheterocyclic agent [11]. Benznidazole treatment does have a major effect on thiol biochemistry within the parasite, and leads to depletion of trypanothione [7]. Because of the central antioxidant role of this major low molecular weight thiol, benznidazole treatment may render *T*. *cruzi* more susceptible to oxidative stress. However, it is implicit that this enhanced susceptibility cannot be mediated *via* a build up of H_2_O_2_ within the ER, as depletion or overexpression of TcAPx has no effect on benznidazole sensitivity.

The *TcAPx* null mutants were able to establish a chronic infection in mice, despite a reduced infection capacity *in vitro*. Although they appeared to produce a slightly shorter acute phase, they displayed similar tissue tropism to wild type parasites, with persistence in the gastro-intestinal tract (particularly in the stomach and the colon) after immune-mediated clearance from most other sites. We have observed a similar pattern in chronic murine infections with the CL-Brener strain of *T*. *cruzi* [19]. It can be implied that TcAPx is not essential for immune evasion and that an oxidative burst that generates high exogenous levels of H_2_O_2_ is not a significant component of the response to this parasite. Deletion of the *Leishmania major* orthologue of *TcAPx* (*LmAPx*) also results in a hypersensitivity to exogenous H_2_O_2_ [33]. However, in that case the null mutants exhibited an enhanced virulence phenotype in the mouse footpad model for cutaneous leishmaniasis. These authors suggest that this may be due to an increased number of “apoptotic” parasites in the null mutant population. The LmAPx protein occurs in the mitochondrion rather than the endoplasmic reticulum and therefore plays a different biological role in *Leishmania* compared to *T*. *cruzi* [14]. Secondly, invasion of *Leishmania* is restricted to professional phagocytic cells where the parasite replicates in the phagolysosome, whereas *T*. *cruzi* can infect both phagocytic and non-phagocytic cell types, and replicates in the host-cell cytoplasm. These factors could account for the differential effects on virulence.

In summary, the data presented here clearly demonstrate that TcAPx is not a suitable target for drug development, since inhibition of its activity would not have a significant effect on parasite virulence or infectivity.

## Supporting Information

S1 Fig *TcAPx* genomic loci are not well conserved in the CL-Brener strain. Map of the genomic environment of the *TcAPx* alleles in the CL-Brener strain of *T*. *cruzi*. TcCL-NEL represents the non-Esmeraldo allele and TcCL-EL indicates the Esmeraldo-like allele. The structure of the locus from Sylvio X10/6 characterised in this study is represented below (labelled TcX10/6). Pseudogenes are denoted by ψ and the double-headed arrow indicates the transcriptional strand-switch region. The open box represents the location of the degenerate *VIPER/SIRE* element in each locus. The protein coding genes shown are: G6PDH: glucose-6-phosphate dehydrogenase, DNAJ: DNAJ-domain containing chaperone, CLPTM1: Cleft-lip and palate transmembrane 1–like protein, TS: *trans*-sialidase.
(TIF)

S2 Fig *TcAPx* cannot be ablated using conventional gene disruption. **A** Map showing the two *TcAPx* disruption constructs and the restriction sites used in analysis (Sp: Spe I, C: Cla I). Blue lines indicate expected restriction fragments from each locus. Grey boxes indicate *T*. *brucei* tubulin RNA processing signals, X indicates sites of recombination. **B** Schematic of the tandem repeat of the *TcAPx-BLA* construct in cell line 7 (panels F and G). Restriction fragments derived from this array are shown in red, the orange box indicates the plasmid-derived junction region absent when the construct is correctly integrated. **C** Southern blot of genomic DNA digested with Cla I and Spe I probed with the *TcAPX* ORF. Lane 1: Wild Type, lane 2: Cells transformed with *PAC* construct, lane 3: Clonal line transformed with both *PAC* and *BLA* constructs. The wild type allele is indicated as fragment 1 in panel A and migrates at ~9 kb. The *PAC* insertion introduces Spe I and Cla I sites into the locus and gives rise to a band of 2 kb, representing the 5’ flanking DNA (fragment 2 in panel A) and a band of ~6.7 kb, representing the 3’ flanking DNA (fragment 3 in panel A). The *BLA* insertion creates a 3’ hybridising band which runs between the wild type and *PAC* disrupted alleles at 7.9 kb (fragment 4 in panel A). To confirm that the wild type locus was still present in this clone, chromosomal DNA was analysed by CHEFE. This blot was probed with the region of *TcAPx* deleted in the targeting constructs (*TcΔAPx*, underneath main blot). Hybridisation in lane 3 confirmed that the cell line was triploid for this locus. The blot was also probed with *PAC* and *BLA* as shown underneath panels D and E. **D** Southern blot of genomic DNA digested with ClaI I and Spe I probed with the *PAC* ORF. Lanes as in C. The 0.8 kb band in lanes 2 and 3 corresponds to the Spe I→Cla I *PAC* gene (fragment 5 in panel A). The weak hybridisation to fragment 4 in lane 3 is due to *T*. *brucei* tubulin intergenic sequences present in the construct from which the probe was isolated which recognise tubulin sequences in the *BLA* construct. **E** Southern blot of genomic DNA digested with ClaI I and Spe I probed with the *BLA* ORF. Lanes as in C. Clone 3 shows hybridisation with the *BLA* probe at the expected size of ~8 kb indicating that the construct has integrated in the correct locus (fragment 4 in panel A). **F** Southern blot of genomic DNA digested with ClaI I and Spe I probed with the *TcAPX* ORF. Lanes 4–7, clonal lines transformed with both *PAC* and *BLA* constructs. In clones 4 and 5, the band corresponding to the original *PAC* insertion has been deleted (expected location marked *) and a novel band of unpredicted size (~3.5 kb, indicated by #) has appeared. Clone 6 shows natural resistance to blasticidin and is untransformed with the second construct. Clone 7 shows expected bands for the wild type *TcAPx* gene (fragment 1) and the *PAC* insertion (fragment 3), however there are intense bands at 0.5 and 1.2 kb, of which the 1.2 kb band hybridises to the *BLA* ORF (panel G, fragment A). These bands are predicted from a tandem array of the input construct replicating episomally (panel B). Unpredicted bands were also observed. **G** Southern blot of genomic DNA digested with ClaI I and Spe I probed with the *BLA* ORF. The blot shows DNA from clone 7 and confirms that the 1.2 kb intense band (panel F) corresponds to the fragment containing the *BLA* gene. Since all clones tested showed the presence of an unmodified wild type *TcAPx* allele, they were not characterised further.
(TIF)

S3 Fig Expression of luciferase does not significantly affect the doubling time of the null mutants compared to the wild type. Parasites were cultured in triplicate in 12 well plates and their growth followed by counting every 48 hours. The doubling time for each cell line was calculated from the exponential growth phase. The chart shows mean doubling time with error bars indicating standard deviation. The table gives the doubling time in hours +/- SD. One way ANOVA indicated no significant difference in doubling time between the parasite lines (*P* = 0.4)
(TIF)

S4 Fig Luciferase activity of wild type and TcΔAPX clones expressing PpyRE9h red shifted luciferase *in vitro*. Luciferase activity was assayed at 610 nM in extracts of mid-log phase epimastigotes (Methods). The assays were carried out on two individual extracts per cell line and each extract was assayed in duplicate. Bars indicate mean luciferase activity per 10^6^ parasites.
(TIF)
