# Supplementary figures and images for: The Trypanosoma cruzi Vitamin C Dependent Peroxidase Confers Protection against Oxidative Stress but Is Not a Determinant of Virulence
Source: PLoS Negl Trop Dis. 2015 Apr 13;9(4):e0003707. doi: 10.1371/journal.pntd.0003707 (PMC4395405; doi:10.1371/journal.pntd.0003707)

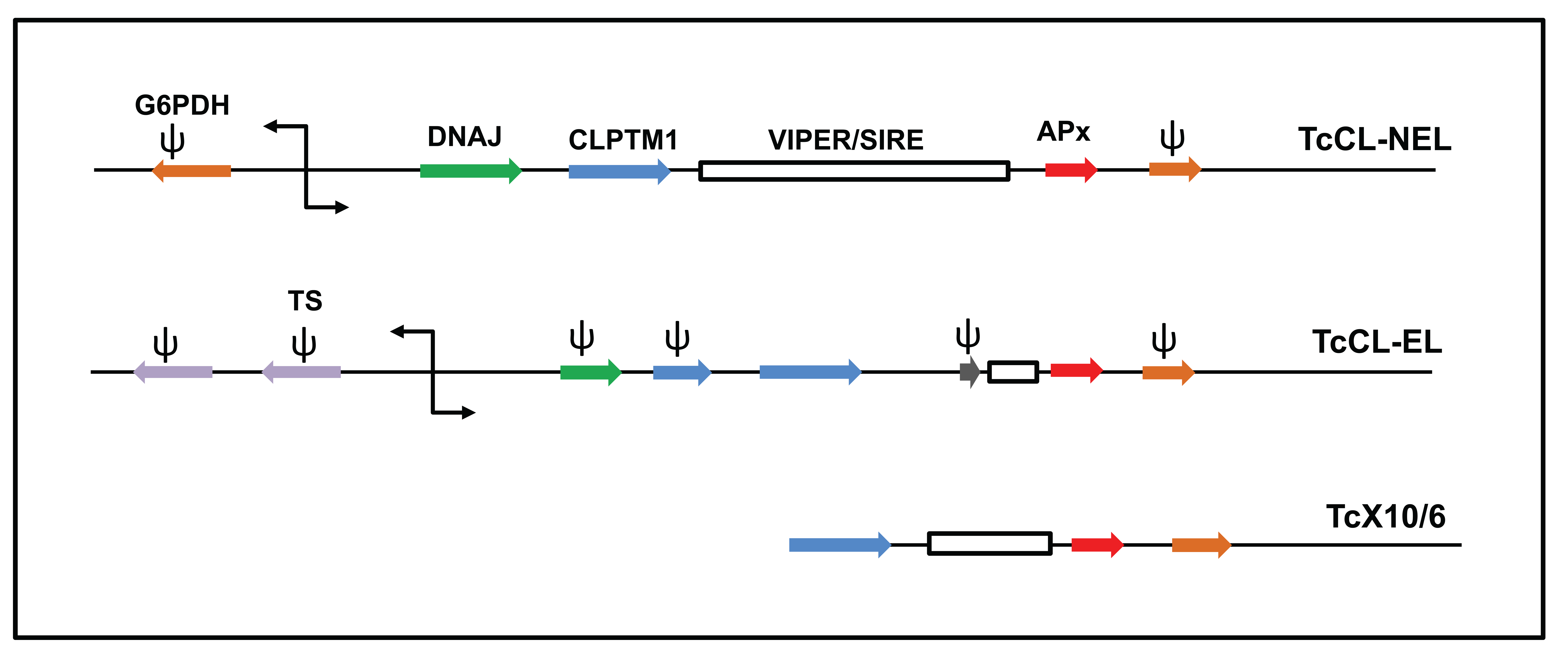

Supplement: S1 Fig — Map of the genomic environment of the TcAPx alleles in the CL-Brener strain of T. cruzi. TcCL-NEL represents the non-Esmeraldo allele and TcCL-EL indicates the Esmeraldo-like allele. The structure of the locus from Sylvio X10/6 characterised in this study is represented below (labelled TcX10/6). Pseudogenes are denoted by ψ and the double-headed arrow indicates the transcriptional strand-switch region. The open box represents the location of the degenerate VIPER/SIRE element in each locus. The protein coding genes shown are: G6PDH: glucose-6-phosphate dehydrogenase, DNAJ: DNAJ-domain containing chaperone, CLPTM1: Cleft-lip and palate transmembrane 1–like protein, TS: trans-sialidase. (TIF) [file pntd.0003707.s001.tif]

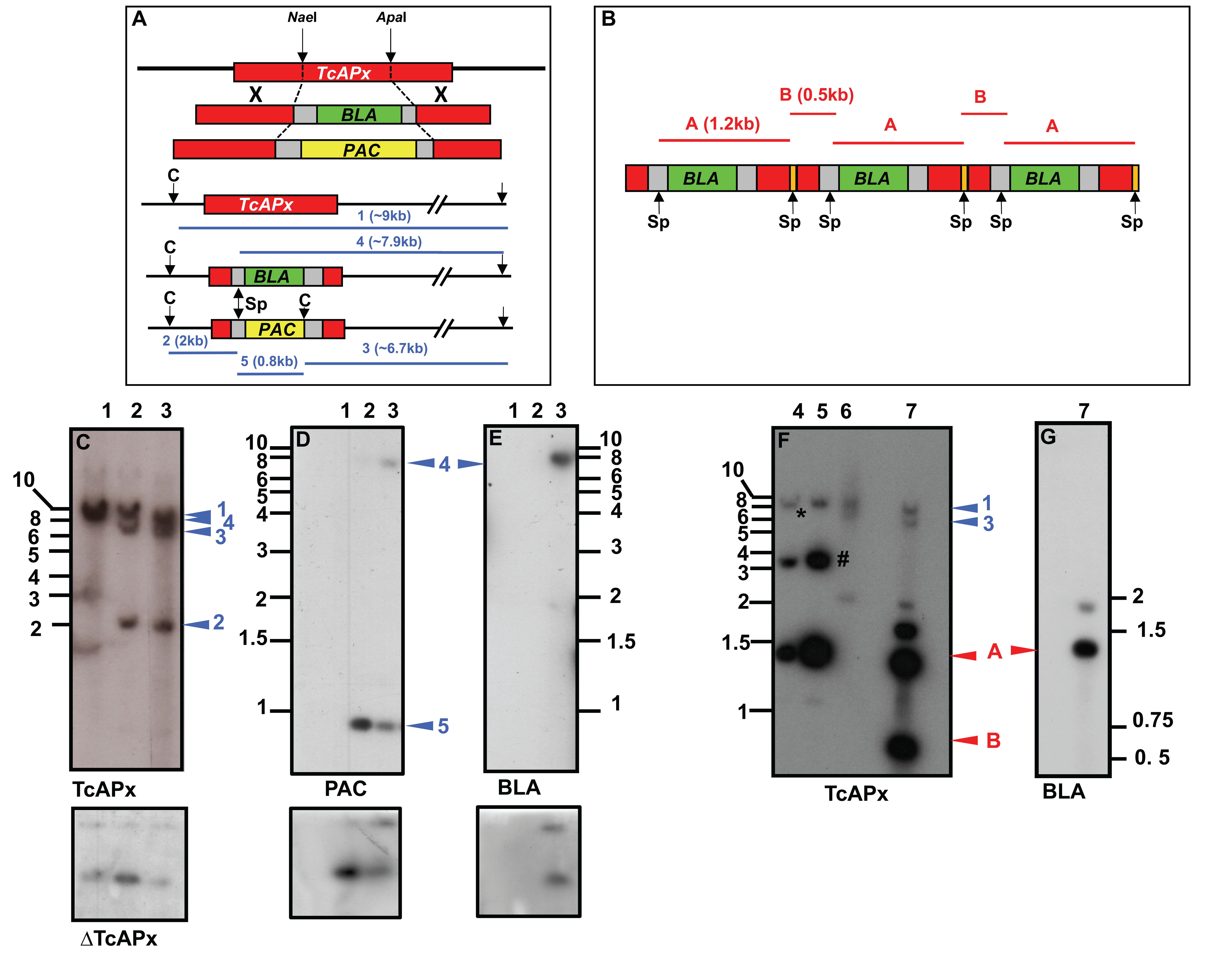

Supplement: S2 Fig — A Map showing the two TcAPx disruption constructs and the restriction sites used in analysis (Sp: Spe I, C: Cla I). Blue lines indicate expected restriction fragments from each locus. Grey boxes indicate T. brucei tubulin RNA processing signals, X indicates sites of recombination. B Schematic of the tandem repeat of the TcAPx-BLA construct in cell line 7 (panels F and G). Restriction fragments derived from this array are shown in red, the orange box indicates the plasmid-derived junction region absent when the construct is correctly integrated. C Southern blot of genomic DNA digested with Cla I and Spe I probed with the TcAPX ORF. Lane 1: Wild Type, lane 2: Cells transformed with PAC construct, lane 3: Clonal line transformed with both PAC and BLA constructs. The wild type allele is indicated as fragment 1 in panel A and migrates at ~9 kb. The PAC insertion introduces Spe I and Cla I sites into the locus and gives rise to a band of 2 kb, representing the 5’ flanking DNA (fragment 2 in panel A) and a band of ~6.7 kb, representing the 3’ flanking DNA (fragment 3 in panel A). The BLA insertion creates a 3’ hybridising band which runs between the wild type and PAC disrupted alleles at 7.9 kb (fragment 4 in panel A). To confirm that the wild type locus was still present in this clone, chromosomal DNA was analysed by CHEFE. This blot was probed with the region of TcAPx deleted in the targeting constructs (TcΔAPx, underneath main blot). Hybridisation in lane 3 confirmed that the cell line was triploid for this locus. The blot was also probed with PAC and BLA as shown underneath panels D and E. D Southern blot of genomic DNA digested with ClaI I and Spe I probed with the PAC ORF. Lanes as in C. The 0.8 kb band in lanes 2 and 3 corresponds to the Spe I→Cla I PAC gene (fragment 5 in panel A). The weak hybridisation to fragment 4 in lane 3 is due to T. brucei tubulin intergenic sequences present in the construct from which the probe was isolated which recognise tubulin sequenc [file pntd.0003707.s002.tif]

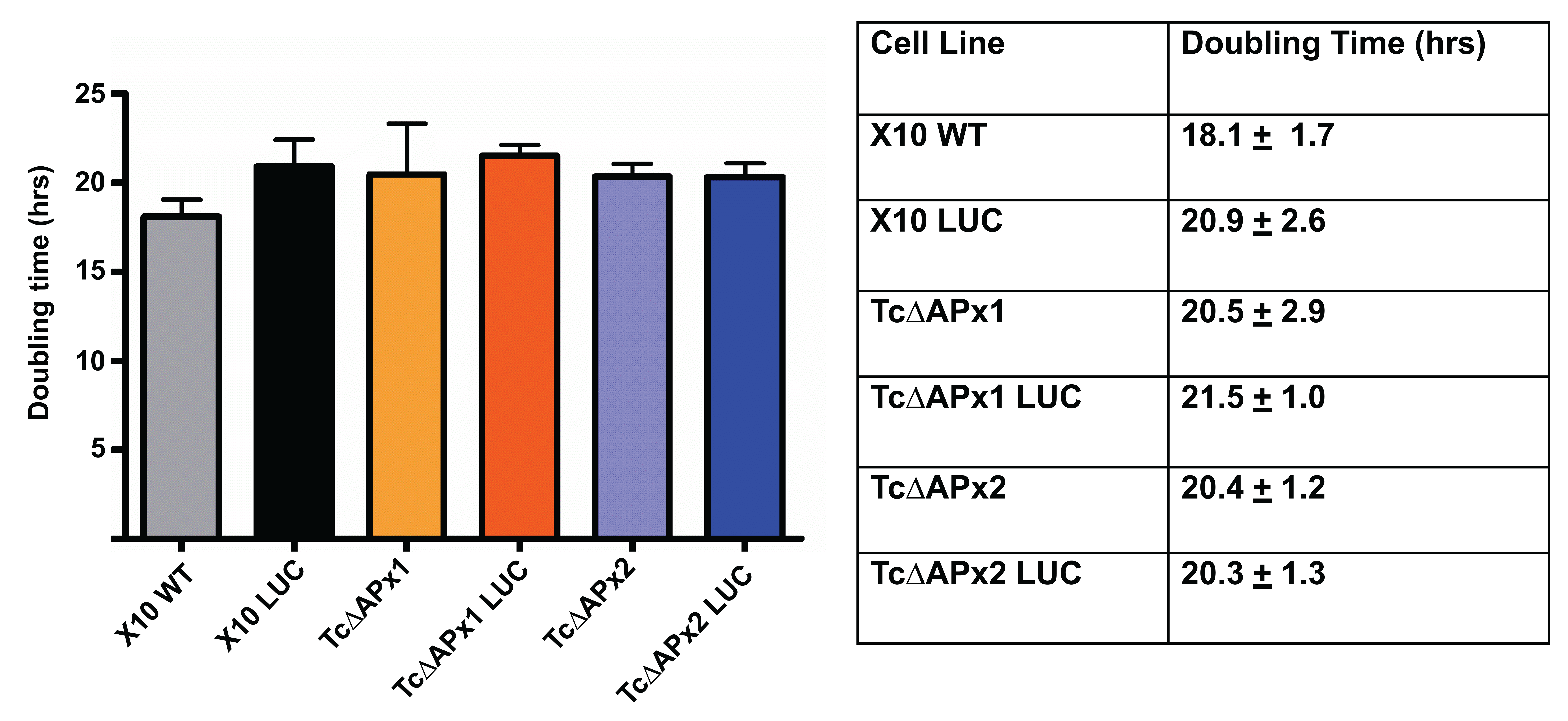

Supplement: S3 Fig — Parasites were cultured in triplicate in 12 well plates and their growth followed by counting every 48 hours. The doubling time for each cell line was calculated from the exponential growth phase. The chart shows mean doubling time with error bars indicating standard deviation. The table gives the doubling time in hours +/- SD. One way ANOVA indicated no significant difference in doubling time between the parasite lines (P = 0.4) (TIF) [file pntd.0003707.s003.tif]

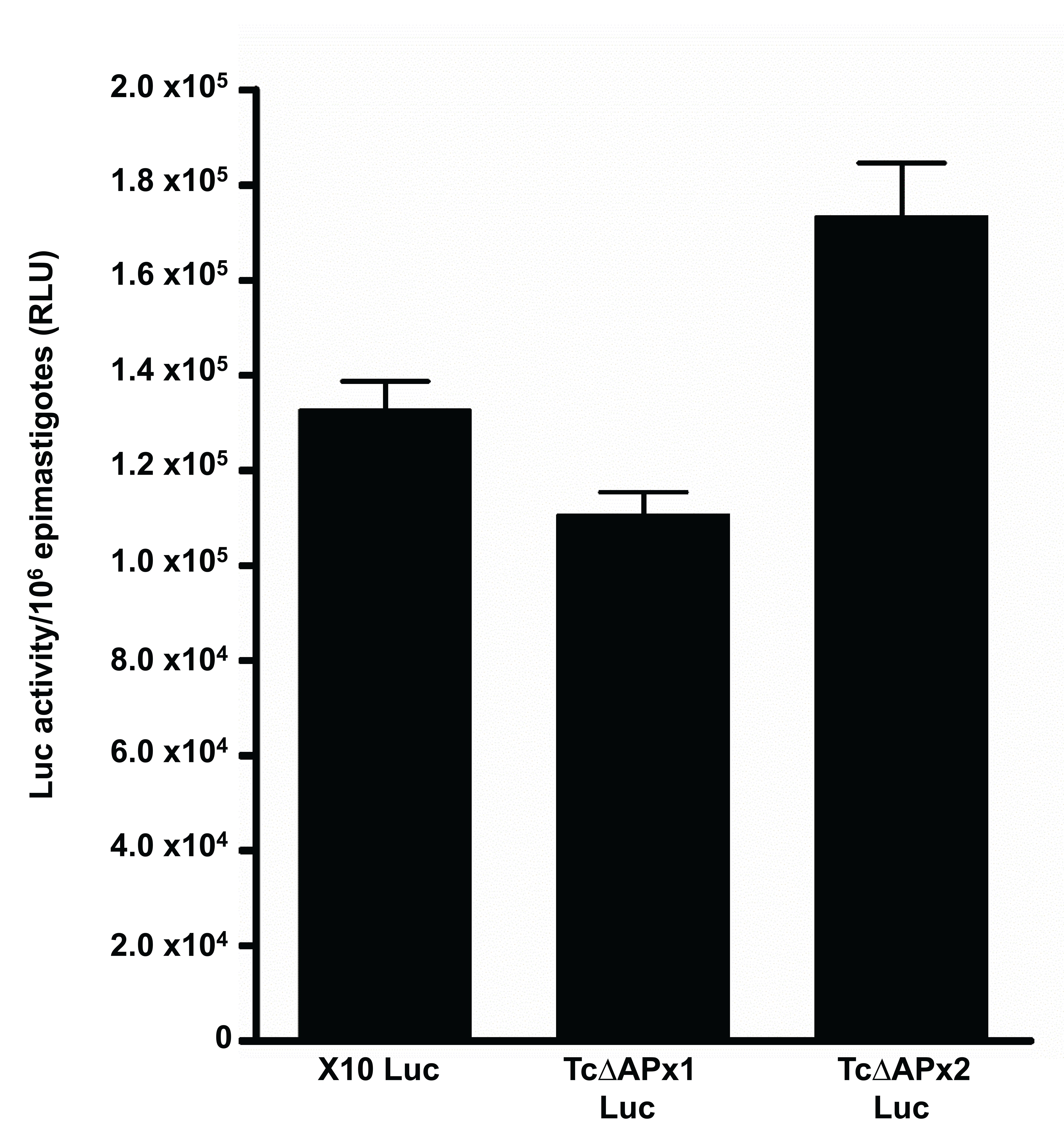

Supplement: S4 Fig — Luciferase activity was assayed at 610 nM in extracts of mid-log phase epimastigotes (Methods). The assays were carried out on two individual extracts per cell line and each extract was assayed in duplicate. Bars indicate mean luciferase activity per 106 parasites. (TIF) [file pntd.0003707.s004.tif]
